# Letter to the Editor on “Mesenchymal stem cells enhance the oncolytic effect of Newcastle disease virus in glioma cells and glioma stem cells via the secretion of TRAIL”

**DOI:** 10.1186/s13287-017-0571-9

**Published:** 2017-06-05

**Authors:** Mingli Liu

**Affiliations:** Department of Microbiology, Biochemistry and Immunology, Morehouse School of Medicine, RW339, 720 Westview Drive SW, Atlanta, GA 30310 USA

I read with great interest the article by Kazimirsky et al. [1] entitled “Mesenchymal stem cells enhance the oncolytic effect of Newcastle disease virus in glioma cells and glioma stem cells via the secretion of TRAIL” (see related article by Kazimirsky et al., http://stemcellres.biomedcentral.com/articles/10.1186/s13287-016-0414-0). The author reports that Newcastle disease virus (NDV)-infected umbilical cord-derived mesenchymal stem cells (MSCs) may provide a novel effective therapeutic approach for targeting glioma stem cells (GSCs) and glioblastoma multiforme (GBM), and for sensitizing these tumors to γ-radiation [1]. The approaches and experimental designs of this work are technically sound. Logically, Kazimirsky et al. concluded a synergistic role of NDV and MSCs in the treatment of glioma. Without any doubt, the work has repeated and strengthened already published work by others.

A couple of additional points can be made on this topic. The findings of Kazimirsky et al. [1] regarding the oncolytic effects on glioma caused by NDV are not novel. The oncolytic effects on glioma were reported as early as 2006 [2]. This observation was subsequently extended in other studies during 2007 to 2015, including the publication by Koks et al. in the *International Journal of Cancer* [3] and an additional three publications [4–6]. In addition, Kazimirsky et al. [1] stated that MSCs enhance the oncolytic effect of NDV in glioma cells via the secretion of TNF-related apoptosis-inducing ligand (TRAIL). In fact, it was reported in 2006 that NVD exerts oncolysis by both intrinsic and extrinsic (TRAIL) caspase-dependent pathways of cell death [7]. In particular, TRAIL-secreting human umbilical cord blood-derived MSCs had been used against intracranial glioma as a gene therapy in 2008 [8]. From the aspect of contributing to biomedical studies, replication research is crucial for the progress of science. However, novelty should be considered as one of the criteria for publication of research works in a journal with a high impact factor such as *Stem Cell Research & Therapy*.

Continuing with this important issue, in an article with 70 references none of the previously published work is discussed or cited by Kazimirsky et al. [1]. As we know, references must be complete and accurate and indicate how the study fits with previous ones in the same field. The reader should be aware of other studies to have a complete picture on this topic.

## Abbreviations

GBM: Glioblastoma multiforme
GSC: Glioma stem cell
MSC: Mesenchymal stem cell
NDV: Newcastle disease virus
TRAIL: Tumor necrosis factor-related apoptosis-inducing ligand
